# Assessing the resilience of HIV healthcare services provided to adolescents and young adults after the COVID-19 pandemic in the city of Beira (Mozambique): an interrupted time series analysis

**DOI:** 10.1186/s12981-024-00621-8

**Published:** 2024-05-09

**Authors:** Roberto Benoni, Francesco Cavallin, Virginia Casigliani, Annachiara Zin, Dara Giannini, Izilda Chaguruca, Vasco Cinturao, Fernando Chinene, Giulia Brigadoi, Daniele Donà, Giovanni Putoto, Carlo Giaquinto

**Affiliations:** 1 Division of Pediatric Infectious Diseases, Department of Women’s and Children’s Health, University of Padua, Padua, Italy; 2 Doctors with Africa CUAMM, Beira, Mozambique; 3 Section of Hygiene, Department of Diagnostics and Public Health, University of Verona, Strada Le Grazie, 8, 37134 Verona, Italy; 4 Independent Statistician, Solagna, Italy; 5 Department of Translational Research and of New Surgical and Medical Technologies, University of Pisa, Pisa, Italy; 6 Section of Operational Research, Doctors With Africa CUAMM, Padua, Italy

**Keywords:** HIV, COVID-19, Healthcare access, Adolescents and young people, Mozambique

## Abstract

**Background:**

The COVID-19 pandemic has put the provision of health services globally at risk. In Sub-Saharan Africa, it had a major impact on HIV services. However, there is a lack of data on the post-pandemic period. This study aims to evaluate the resumption of HIV services and retention in care for adolescents and young people in the period following the COVID-19 pandemic.

**Methods:**

A retrospective cohort study was conducted using interrupted time series analysis. Three periods were considered: pre-pandemic (form June 2019 to March 2020), pandemic (form April 2020 to March 2022) post-pandemic (from April 2022 to March 2023). Six outcome measures were considered: number of outpatient visits, HIV tests, HIV positivity ratio, the antiretroviral treatment (ART) non-adherence ratio, recall ratio, and the return ratio for adolescent and young adults on ART.

**Results:**

During the study period, 447,515 outpatient visits and 126,096 HIV tests were recorded. After a reduction at the beginning of the pandemic period, both visits and tests increased during the pandemic (p < 0.05) and decreased in the post-pandemic (p < 0.05), recovering the pre-pandemic trends. The HIV positivity ratio slightly decreased from 3.3% to 1.7% during the study period (p < 0.05). The ART non-adherence ratio decreased from 23.4% to 2.4% throughout the study period (p < 0.05), with a drop at the beginning of the post-pandemic period (p < 0.05). The recall ratio increased during the study period (p < 0.05) with a drop at the beginning of the pandemic and post-pandemic periods (p < 0.05). The return ratio decreased at the beginning of the pandemic (p < 0.05) but returned to the pre-pandemic ratio in the post-pandemic period.

**Conclusions:**

The post-pandemic values of the investigated outcomes were comparable to pre-pandemic period, or even improved. Differently from other services, such as the community activities, that have been severely affected by COVID-19 pandemic, the HIV service system has shown resilience following emergency situation.

## Introduction

The onset of the COVID-19 pandemic, caused by severe acute respiratory syndrome coronavirus 2 (SARS-CoV-2), was expected to be an extremely worrying event for the African continent. However, the direct health impact of COVID-19 was milder than anticipated [1]. The limited spread and mortality from SARS-CoV-2 may have been driven by several factors, including poor testing levels, policy makers and socio-epidemiological characteristics of the population. When evaluating these data, another issue to consider is the large degree of uncertainty associated with COVID-19-directed mortality which depends on different mortality modelling approaches [2]. When the World Health Organization (WHO) declared the end of COVID-19 as a public health emergency on 5 May 2023, 233,417 cases were confirmed in Mozambique, with 2243 deaths [3]. On the contrary, the indirect effects of the pandemic have heavily afflicted Sub-Saharan Africa (SSA) in terms of food insecurity, lack of medical supplies, loss of income and livelihoods [3]. Particularly affected was access to health services at different levels: Human Immunodeficiency Virus (HIV) care, treatment for malaria and tuberculosis, and maternal and child health services [4]. In SSA, the lockdown was associated with an estimated 47.6% decrease in HIV testing in April 2020 and a 46.2% decrease in Antiretroviral Therapy (ART) initiation in the first week after the COVID-19 pandemic lockdown [5]. Availability of ART and consistent adherence are the key pillars to sustain viral suppression and thus prevent disease outbreaks and reduce HIV morbidity, mortality, and transmission in all age groups [6]. Hence, it was estimated that HIV-related deaths will increase by up to 10% over the next five years due to ART interruptions caused by COVID-19 in low- and middle-income countries (LMICs) [7].

In a region such as SSA accounting for 77.5% of the new HIV infection in the population aged 15–24 years in 2022, it is particularly important to monitor the impact of COVID-19 pandemic on HIV health services [8]. Eastern and Southern Africa (ESAR) account for 53.3% (20.8 million/39.0 million) of global people living with HIV (PLHIV) [8]. In 2021, Mozambique registered 94,000 new cases of HIV and became the second country in SSA with the highest number of infections [9]. In the same year, nearly 2 million PLHIV (5.1% of global PLHIV) were estimated to live in the country [9].

Most ESAR countries suffered major reductions in access to HIV services and adherence to ART following the COVID-19 pandemic, especially for the adolescents and youth [10]. Available ESAR data showed a drop ranging from 37.4% (Ethiopia) and 36.1% (Malawi) to 47.6% (South Africa) in monthly HIV testing after the beginning of the COVID-19 pandemic [11–13].

Despite the extensive literature on the disruption of HIV health services caused by the lockdown during the pandemic, little is known about their status after the end of the restrictive measures. Hence, this study aimed to evaluate the resumption of HIV services and retention in care for adolescents and young people in the period following the end of the COVID-19 pandemic in a low-resource setting. In addition, potential differences in service provision according to the location of the health center (central or peripheral) were explored.

## Methods

### Study design and ethical approval

A retrospective, observational, multicenter study was conducted to evaluate the resumption of services after the declaration of the end of State of Public Calamity for the COVID-19 pandemic in Mozambique. The research was performed following the ethical standards of the 1964 Declaration of Helsinki and was approved by the Comité Interinstitucional de Bioética para Saúde (CIBS) (protocol number 057/CIBS/2022).

### Setting and pandemic phases

The city of Beira is located in the province of Sofala, in the central area of Mozambique. It has an estimated population of 2,528,442, of which 897,467 (35.5%) are aged between 10 and 24 years [14]. For this age group, the government of Mozambique has a special service within the health centers (HC) that provides education, prevention, and treatment for adolescents and young adults (AYA) called “Adolescent and youth friendly services” (*Serviços amigos dos adolescentes e jovens*—SAAJ). Seven SAAJ of Beira district, where the Non-Governmental Organization (NGO) Doctors with Africa CUAMM works, were selected. Of these, four SAAJ (Ponta Gea, Munhava, Hospital Central de Beira—HCB, Macurungo) are in a central and three (Inhiamizua, Mascarenha, Nhaconjo) in a peripheral area.

SAAJ provides assistance to people aged between 10 and 24 years ranging from sexual and reproductive health counselling to prevention and treatment of HIV and sexually transmitted infections (STIs). In addition to these services, a recall intervention is provided for PLHIV who discontinue ART. They are defined according to the Mozambican HIV guidelines as all persons who discontinued ART within a period of 5 to 59 days; after 60 days, they are considered lost to follow-up [15]. The recall intervention involves a maximum of three phone calls, held by community health workers, and then, if unsuccessful or the person is unreachable (and if they have given their consent), they receive a home visit. During this contact (telephone or face-to-face), the community health worker provides brief counselling to motivate the person to come back to treatment.

The first case of COVID-19 in Mozambique was reported on 22 March 2020. The declaration of the State of Emergency occurred on 30 March 2020 with the adoption of several prevention and control strategies. These included school closures, prohibition of public and sporting events, mandatory wearing of face masks in public places, curfews, social distancing, and mandatory quarantine for travelers from international travel [16]. It was converted into State of Calamity in September 2020, with an easing of the restriction measures, and it lasted until April 2022, when its end was declared [16].

### Data source

Data were collected routinely monthly by the clinical staff of the HCs aiming at providing reports for local and national health authorities. These data were available at aggregate-level. Data collection at the HC level continued during the study period with the support of the NGOs working in Mozambique and the coordination of the Ministry of Health. Data quality was assured through monthly review by a monitoring and evaluation officer and quarterly data discussions with the staff involved in data collection.

### Study periods

The overall study period (from 1 September 2019 to 31 March 2023) was divided in three periods of interest: the before-pandemic period (from 1 September 2019 to 31 March 2020; the pandemic period (from 1 April 2020 to 31 March 2022) and the post-pandemic period (from 1 April 2022 to 31 March 2023). Data between March and September 2019 were not available due to the cyclone Idai that hit the city of Beira on 14 March 2019 [17]. All data were extracted from monthly HC records by a researcher who was not involved in any clinical activities.

### Outcome measures

Two sets of outcomes were considered. The first set referred to the general population aged 10–24 years old and included the number of outpatient visits, HIV tests and the HIV positivity ratio among individuals attending health facilities. The second set referred specifically to PLHIV in the same age group and included non-adherence, recall, and return ratio.

The non-adherence ratio was estimated by a proxy indicator that included the percentage of PLHIV who did not pick up ART out of the total PLHIV on treatment; the recall ratio was the number of non-adherent PLHIV, as defined above receiving a re-engagement invitation out of the total non-adherent PLHIV; the return ratio was calculated as the number of recalled persons who returned to treatment.

### Statistical analysis

Interrupted time series modelling was applied to monthly data of outcome measures, and the resumption of HIV health services was evaluated by assessing the changes in level and slope of each time series [18]. Since before-pandemic period and pandemic periods included different subset of months, we anticipated some contribution of the seasonality on the observed variations between the periods, without associations with the pandemic itself. The restricted comparison of similar subset of months in the periods was ruled out because it would have reduced the volume of data and limited the estimation capability of the time trends. As the indirect impact of the pandemic could be assessed by the variation in the trend component of the time series, trend and seasonal components of each time series were estimated separately, and the analysis of each outcome measure was performed on the trend component [18]*.* The analysis employed linear mixed effect models (for outpatient visit and HIV tests) and generalized linear mixed effect models using the beta distribution family (for HIV positivity ratio, non-adherent ratio, recall ratio and return ratio). The models included the time (from September 2019 to March 2023), the period (pre-pandemic, pandemic, and post-pandemic) and the interaction term time * period, with the HC incorporated as random effect. A sensitivity analysis explored the possible indirect effect of the location of the HC (central or peripheral) by adding a three-terms interaction time * period * location in the models. All tests were 2-sided and a p < 0.05 was considered statistically significant. Statistical analysis was performed using R 4.3 (R Foundation for Statistical Computing, Vienna, Austria) [19].

## Results

### Outcome measures for the general population

**Fig. 1:**
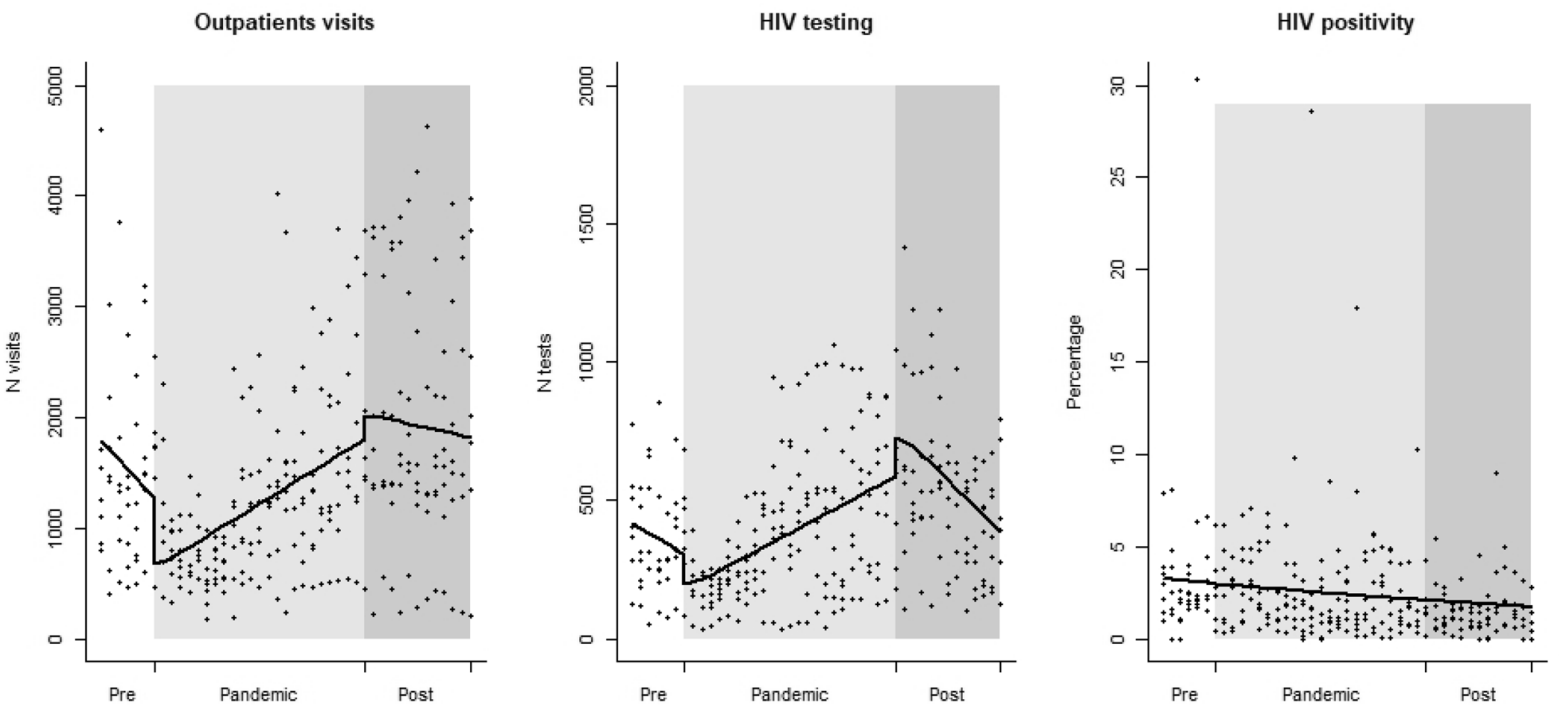
Interrupted time series of outpatient visits, HIV testing and HIV positivity ratio at seven SAAJs in Beira (Mozambique) between September 2019 and March 2023. White background: before-pandemic period (September 2019 to March 2020); silver background: pandemic period (April 2020 to March 2022); grey background: post-pandemic period (April 2022 to March 2023). Line: predicted trend based on the regression model

During the study period, the HCs recorded a total of 447,515 outpatient visits and 126,096 HIV tests, with an average HIV positivity ratio of 2.4%. Figure 1 shows the overall trends in outpatient visits, HIV tests, and HIV positivity ratio. Average monthly outpatient visits decreased by 597 visits at the beginning of the pandemic (p < 0.0001) then increased by 49 visits/month during the pandemic (p = 0.0003); after the pandemic, the trend inverted (p = 0.0001) and was not statistically different when compared to the pre-pandemic (p = 0.09) (Fig. 1). Similarly, average monthly HIV tests decreased by 108 tests at the beginning of the pandemic (p < 0.0001) then increased by 17 tests/month during the pandemic (p = 0.005); after the pandemic, the trend inverted (p < 0.0001) and was not statistically different when compared to the pre-pandemic (p = 0.35) (Fig. 1). On the other hand, the average HIV positivity ratio slightly decreased from 3.3% to 1.7% during the study period (p < 0.0001), without any indirect effects of the pandemic (Fig. 1). Full details of the analysis were reported in Table 1.

**Table 1:** Results from the interrupted time series analysis

Variable	Outpatient visits		HIV tests		HIV positivity ratio		Non-adherent ratio		Recall ratio		Return ratio	
	Coef (SE)	p-value	Coef (SE)	p-value	Coef (SE)	p-value	Coef (SE)	p-value	Coef (SE)	p-value	Coef (SE)	p-value
Time	− 86 (36)	p = 0.020	− 19 (12)	p = 0.130	− 0.02 (0.003)	p < 0.001	− 0.03 (0.006)	p < 0.001	0.06 (0.01)	p < 0.001	− 0.004 (0.005)	p = 0.420
Period (pre-pandemic)												
Pandemic	− 1591 (203)	p < 0.001	− 377 (70)	p < 0.001	Not in the model		− 0.16 (0.13)	p = 0.210	− 1.22 (0.23)	p < 0.001	− 0.51 (0.13)	p < 0.001
Post-pandemic	714 (637)	p = 0.260	1282 (219)	p < 0.001	Not in the model		− 0.90 (0.25)	p < 0.001	− 1.88 (0.38)	p < 0.001	Not in the model	
Time * period (pre-pandemic)												
Time * pandemic	135 (37)	P < 0.001	36 (12)	p = 0.005	Not in the model		Not in the model		Not in the model		Not in the model	
Time * post-pandemic	68 (40)	p = 0.090	− 12 (13)	p = 0.350	Not in the model		Not in the model		Not in the model		Not in the model	

Pre-pandemic period (September 2019 to March 2020); pandemic period (April 2020 to March 2022); post-pandemic period (April 2022 to March 2023)

*Coef* regression coefficient, *SE* standard error, Model selection was performed using the Akaike Information Criterion (AIC)

### Outcome measures for PLHIV

**Fig. 2:**
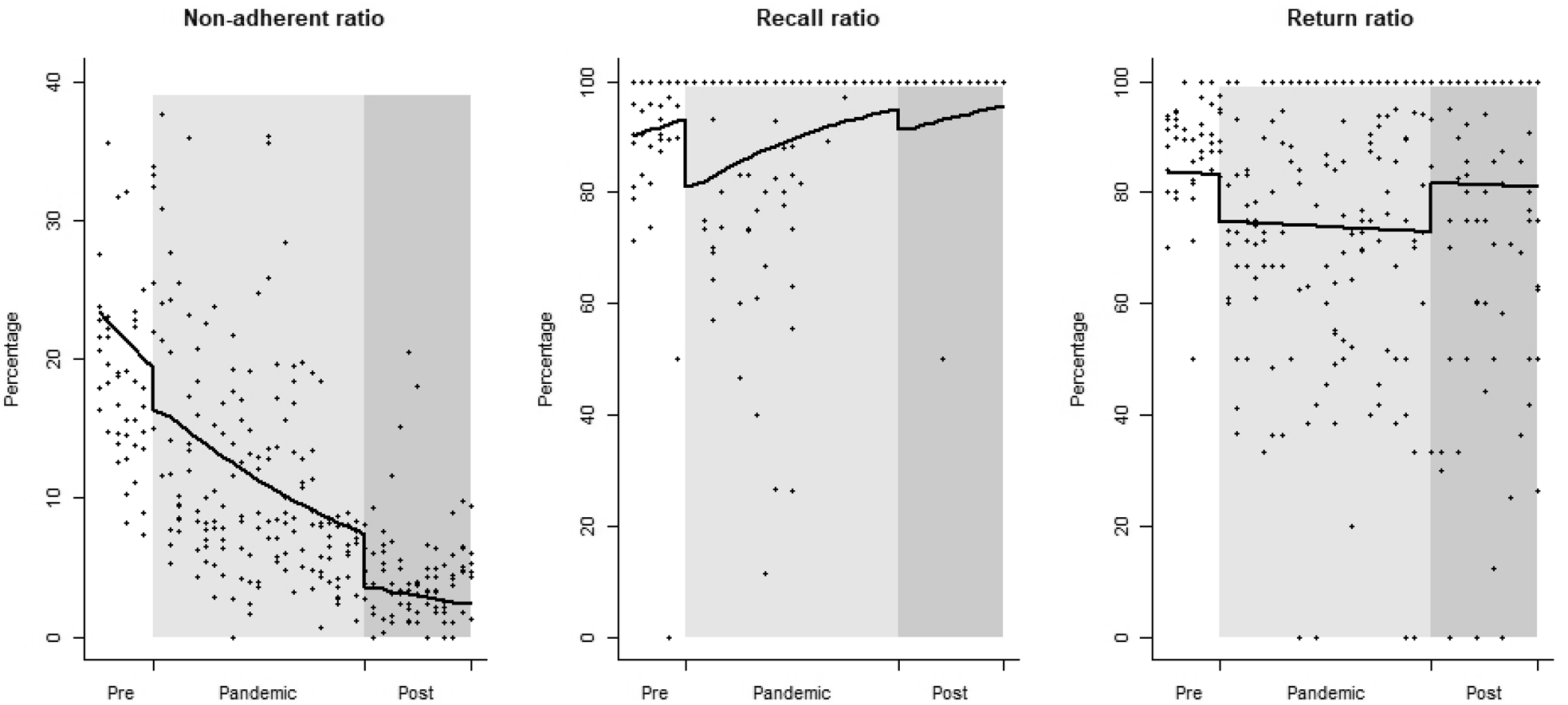
Interrupted time series of non-adherent ratio, recall ratio, and return ratio at seven SAAJs in Beira (Mozambique) between September 2019 and March 2023. White background: before-pandemic period (September 2019 to March 2020); silver background: pandemic period (April 2020 to March 2022); grey background: post-pandemic period (April 2022 to March 2023). Line: predicted trend based on the regression model

During the study period, the HCs recorded an average non-adherent ratio of 8.2%, an average recall ratio of 95.3% and an average return ratio of 79.2%. Figure 2 shows the overall trends in non-adherent ratio, recall ratio, and return ratio. Average non-adherent ratio decreased from 23.4 to 2.4% during the study period (p < 0.0001), with a significant drop at the beginning of the post-pandemic period (p = 0.0003) (Fig. 2). Average recall ratio increased from 90.2 to 95.5%, with a significant drop at the beginning of the pandemic period (p < 0.0001) which affected also the post-pandemic period (p < 0.0001) (Fig. 2). Average return ratio did not show any significant trend over time (p = 0.42) but decreased from 83.2 to 74.7% at the beginning of the pandemic period (p = 0.0001), before returning to pre-pandemic figures (Fig. 2). Full details of the analysis are reported in Table 1.

### Sensitivity analysis on the effect of the location of the HC

When exploring the possible indirect effect of the location (central HCs vs. peripheral HCs), the sensitivity analysis did not show any statistically significant association between the location and the outpatient visits (pandemic period: p = 0.17; post-pandemic period: p = 0.40), HIV testing (pandemic period: p = 0.43; post-pandemic period: p = 0.26), HIV positivity ratio (pandemic period: p = 0.70; post-pandemic period: p = 0.98), non-adherence (pandemic period: p = 0.68; post-pandemic period: p = 0.66), recall ratio (pandemic period: p = 0.88; post-pandemic period: p = 0.69), or return ratio (pandemic period: p = 0.62; post-pandemic period: p = 0.31).

## Discussion

This study explored the resumption of HIV services for AYAs in the period after the end of the state of emergency following the COVID-19 pandemic in Mozambique. Both the number of outpatient visits and HIV testing showed a downward trend compared with the steady increase during the COVID-19 pandemic returning to values comparable with the pre-pandemic period. Throughout the study period, the HIV positivity ratio decreased, reaching 1.7% in the post-pandemic period. Similarly, the non-adherence ratio decreased across the three periods, reaching the lowest value in the post-pandemic period. The recall ratio increased across the three periods, while the return ratio did not show any significant trend despite a decrease during the pandemic period. There was no evidence that the location influenced the indirect impact of the pandemic on the outcome measures.

As already highlighted in the literature, the COVID-19 pandemic had a negative indirect impact on the provision of HIV services for PLHIV in many different countries [20]. In SSA, the major impact occurred between the second and third quarter of 2020 with a reduction of 33.5% and 30.8% in number of HIV test and new ART initiations, respectively, in adolescents aged 10–19 years [21]. On the other hand, an increase from 78.5% to 80.5% in viral load suppression was observed between second and third 2020 quarters [21].

According to our results, both the number of HIV tests and the ART pick up, a proxy measure of adherence, showed an ameliorating trend during the two years of the pandemic, although the HIV tests experienced an initial decline at the beginning of the pandemic. This is in line with findings in other studies where ART services were prioritised and generally maintained even during the early stages of the pandemic while HIV testing was more heavily affected [5, 22]. The constant ameliorating trend found in the present study during the pandemic period for the number of outpatient visits and tests, and for non-adherence and recall ratio could be also due to the fact that the most strict measures were taken during the first wave of the COVID-19 pandemic in Mozambique, similarly to other countries in SSA, while during the second wave, despite its greater severity, there was not the same public health response [23].

In the Fourth round of the global pulse survey on continuity of essential health services during the COVID-19 pandemic conducted by the WHO between November 2022 and January 2023, a general recovery of services was reported in this quarter [24]. In particular, the percentage of disruption of services compared to the pre-pandemic period decreased from 48% in the first quarter of 2021 to 24% in the quarter analyzed for HIV testing and from 32 to 8% for the continuation of established ART [24]. Based on these data, not only there was evidence of a reduction in service disruption, but some countries also experienced an improvement in some HIV services. In particular, an increase over pre-pandemic levels in adherence to ART and in the number of HIV tests was reported by 15% and 9% of the countries surveyed, respectively [24]. Similarly, our findings showed a reduction in the non-adherence rate at the beginning of the post-pandemic period. Both the number of outpatients visits and the number of tests, however, showed a downward trend in the post-pandemic period compared to the pandemic one, although not different from the pre-pandemic period. This could be attributed to a rebound effect after the initial drop at the beginning of the pandemic. Nevertheless, this trend should be closely monitored to avoid losing the positive results obtained during the pandemic. The 2023 executive summary by the Joint United Nations Program on HIV/AIDS (UNAIDS) shows that a total of US$ 20.8 billion was allocated for HIV programs in low- and middle-income countries in 2022. This is 2.6% lower than the funds allocated in 2021 and much lower than the estimated US$ 29.3 billion needed [25]. The provision of adequate economic resources and funding for HIV programs is crucial to maintaining positive trends in the health services delivery. Indeed, although the COVID-19 pandemic has brought a partial increase in investments in the health sector, this must be maintained and adapted to be sustainable and to support health services where it is needed such as HIV services in SSA countries [26]. Maintaining the necessary funds after the COVID-19 pandemic in these services would have a triple beneficial effect by improving the health, social and economic gains of the countries in a virtuous circle [27]. Especially allocating more economic resources to community-based programs is believed to be essential to achieve the 95-95-95 goals for HIV [27].

Among the HIV services most affected by the COVID-19 pandemic along with the number of HIV tests were community programs, such as community support groups, patient tracing activities and outreach activities [28]. The data from our study showed how re-engagement activities were heavily affected by the pandemic, with a decrease compared to the pre-pandemic period, and even at the beginning of the post-pandemic period, the recall ratio made by community health care workers was still at a lower level than in the pre-pandemic period.

The disruption of community activities and testing at the very beginning of the pandemic period may also explained the decreasing positivity rate observed during the study period. Community-based testing is a key pillar for reaching population groups at high risk of HIV. The systematic review on 16 PEPFAR-supported countries found a similar slight decrease from 1.9% before COVID-19 to 1.7% during COVID-19 [21]. The discontinuation of outreach and testing services in communities due to the pandemic suppressed many of the case-finding strategies for key populations. Among these there may be the AYAs who were most affected by the reduction in HIV testing and diagnoses in SSA [29].

In LMICs, the adaptation of HIV services mainly relayed on differentiated service delivery (DSD), in particular for ART services [30]. In Mozambique, managers and providers from the Ministry of Health acknowledged a positive effect of the COVID-19 pandemic, as it loosened eligibility criteria for enrolment in fast-track and 3-months ART dispensing [31]. DSD has the advantage of being a person-centred approach that simplifies and adapts HIV services throughout the cascade, reducing unnecessary burdens on the health system [31]. This model not only was effective in maintaining HIV services but also had the advantage of limiting the impact of COVID-19 infection in a fragile and at-risk population such as PLHIV [32]. Indeed, seroprevalence studies showed no significant differences in SARS-CoV-2 infection probability between the general population and PLHIV [33]. Throughout the pandemic period, HIV services were never interrupted and, as in other countries, Mozambique prioritised healthcare workers, as well as other at-risk groups, for vaccination for SARS-CoV-2 [34].

In the present study, no differences in HIV service delivery were found based on the HC being located in a central or peripheral area within an urban setting. This may be due to the fact that HCs located in rural areas were not included in the study, as these areas showed the greatest fragility and difficulties in HIV service delivery as they are also highly reliant on community-based activities [28, 35]

This study has some limitations. First, the pre-COVID period encompassed a time frame of less than one year and following cyclone Idai, so it may not represent a standard of normality reference for HIV services. Second, data on testing and outpatient visits were collected on an aggregate level, so we could not assess how many new people were reached by HIV services during the reporting period nor explore possible differences in access to care based on sex. Finally, data on HIV disease stage and viral load were not collected and analysed, which would also have enabled an assessment of the clinical status of PLHIVs over the three periods included in this study.

## Conclusions

This study showed that the trend of the six outcome measures got back comparable to the pre COVID-19 period in the post pandemic one. The non-adherence ratio had a stable downward trend, probably thank to the improvement of the differentiated delivery of the service, through the extension of the ART delivery and the application of less restrictive criteria. Despite some drops in the early stage, almost all outcome measures showed improvement during the two-year pandemic period, except for the return ratio that nevertheless returned to pre-pandemic levels in the post-pandemic one. The HIV service system proved adaptability and resilience and can serve as a model for other emergency situations, related to new infectious outbreaks, climate change and extreme natural events.

## Data Availability

The datasets generated and/or analysed during the current study are available from the corresponding author on reasonable request.

## Abbreviations

ART: Anti-retroviral therapy
AYA: Adolescents and young adults
DSD: Differentiated service delivery
ITS: Interrupted time series
HC: Health Centres
LMIC: Low Middle-Income Countries
PLHIV: People living with HIV
WHO: World Health Organization
